# Catalase-peroxidase StKatG2 from *Salinicola tamaricis*: a versatile Mn(II) oxidase that decolorizes malachite green

**DOI:** 10.3389/fmicb.2024.1478305

**Published:** 2024-11-05

**Authors:** Mengyao Ding, Wenjing Wang, Zhenkun Lu, Yuhui Sun, Xinzhen Qiao, Meixue Dai, Guoyan Zhao

**Affiliations:** College of Life Science, Shandong Normal University, Jinan, China

**Keywords:** catalase-peroxidase, Mn(II) oxidases, malachite green, decolorization ability, enzymic activity

## Abstract

Manganese (Mn) oxidation processes have garnered significant attention recently due to their potential for degrading organic pollutants. These processes are primarily catalyzed by Mn(II) oxidases. *Salinicola tamaricis* F01, an endophytic bacterium derived from wetland plants, has demonstrated Mn(II)-oxidizing capacity. In this study, a catalase-peroxidase, StKatG2, was cloned and overexpressed in *Escherichia coli* from the strain F01. The purified recombinant StKatG2 exhibited Mn(II)-oxidizing activity with *K*_m_ and *K*_cat_ values of 2.529 mmol/L and 2.82 min^−1^, respectively. Optimal catalytic conditions for StKatG2 were observed at pH 7.5 and 55°C, with 45.1% activity retention after an 8-h exposure to 80°C. The biogenic manganese oxides produced by StKatG2 exhibited mixed-valence states with Mn(II), including Mn(III), Mn(IV), and Mn(VII). Furthermore, StKatG2 demonstrated superior decolorization efficiency for malachite green (MG), achieving decolorization rates of 73.38% for 20 mg/L MG and 60.08% for 50 mg/L MG, while degrading MG into 4-(dimethylamino)benzophenone. Therefore, the catalase-peroxidase StKatG2 exhibits multifunctionality in Mn(II)-oxidizing activity and has the potential to serve as an environmentally friendly enzyme for MG removal.

## 1 Introduction

Malachite green (4-[(4-dimethylaminophenyl)-phenyl-methyl]-*N*, *N*-dimethyl aniline; MG) is a synthetic triarylmethane dye used in the food industry, medical disinfection, and for coloring various materials like leather and fabrics (Rashtbari and Dehghan, 2021; Sahu and Poler, 2024). However, MG in wastewater poses serious health risks, including teratogenic, carcinogenic, and mutagenic effects (Ng et al., 2013; Sartape et al., 2013; Shivaraju et al., 2017; Rout and Jena, 2021). MG is highly stable and resistant to degradation due to its three benzene rings (Xu et al., 2006). Only a few enzymes, such as triphenylmethane reductase (TMR) (Wang et al., 2012; Shang et al., 2019), manganese peroxidase (Yang et al., 2016), laccase (Casas et al., 2009), and tyrosinase (Shedbalkar et al., 2008), can degrade or modify MG. However, incomplete enzymatic conversion can increase the toxicity of MG by-products (Zhou et al., 2019). Moreover, the limited natural production and instability of some enzymes under high temperatures and ionic conditions restrict their applicability (Martins et al., 2015). Therefore, it is crucial to develop new and viable enzymatic approaches for the degradation of these dyes.

Biogenic manganese (Mn) oxidation, driven by Mn(II)-oxidizing microorganisms, is an effective method for degrading organic pollutants (Tran et al., 2018; Li et al., 2021; Liu et al., 2023). These microorganisms use enzymatic oxidases to produce biogenic manganese oxides (BioMnOx), which have high redox potential and strong adsorptive capacity. BioMnOx can oxidize various organic pollutants, such as phenols, chlorinated phenols, chlorinated anilines, atrazine, and various inorganic pollutants. For example, a Mn(II)-oxidizing microbial community in rivulet sediment, comprising core microbes such as *Sphingobacterium* and *Bacillus*, effectively decolorizes dyes (Wan et al., 2020). Moreover, the combination of the Mn(II)-oxidizing bacterium *Pantoea eucrina* SS01 and its BioMnOx efficiently and sustainably degrade MG (Sun et al., 2021). Previous research shows that Mn(II)-oxidizing bacteria use Mn(II) oxidases, which include Multi-Copper Oxidases (MCOs), Mn peroxidases (MnPs), and Mn catalases, for Mn(II) oxidation (Zhou and Fu, 2020). These Mn oxidases show minimal sequence similarities and are predominantly large enzymes with challenges in heterologous gene expression (Zhou and Fu, 2020). Their role in dye degradation is not well-explored. Among these, only MnP has been identified as a viable system for Mn(II)-dependent reactions, enabling the *in vitro* degradation of MG and recalcitrant polymeric dyes (Moreira et al., 2001; Champagne and Ramsay, 2005; Saravanakumar et al., 2013).

Recently, a catalase-peroxidase designated as StKatG (referred to as StKatG1 in this study) from *Salinicola tamaricis* F01, an endophytic Mn(II)-oxidizing bacterium isolated from the halophyte *Tamarix chinensis* Lour, has been identified as a distinct clade of bacterial Mn(II) oxidases (Zhao et al., 2023). This enzyme is capable of converting Mn(II) into biogenic Mn oxides, including MnO_2_, Mn_3_O_4_, and Mn oxalate (Zhao et al., 2023), which may hold potential applications in the adsorption and oxidation of organic pollutants. Beyond its Mn(II) oxidizing capabilities, catalase-peroxidase is a versatile enzyme with Mn(II) oxidizing, catalase, peroxidase, and peroxynitritase activities. It can catalyze multiple reactions, such as hydroxylation, epoxidation, halogenation of C-H bonds, and transformations of aromatic groups and biophenols (Takio et al., 2021; Shen and Wang, 2024). Although limited information exists regarding the application of catalase-peroxidase in the degradation of organic pollutants, a thermostable catalase-peroxidase from *Thermobacillus xylanilyticus* has shown efficacy in oxidizing small aromatic compounds derived from lignins (Fall et al., 2023). Further research into the potential enhancement of catalase-peroxidase activity by Mn(II) could be valuable, as it may expand the enzyme’s applicability in organic pollutant degradation.

The study aimed to investigate the decolorization capability of the catalase-peroxidases toward MG. *Salinicola tamaricis* F01 contains two catalase-peroxidases, both of which demonstrated the ability to decolorize MG, with StKatG2 exhibiting superior efficacy. Previous research has identified StKatG1 as a Mn(II) oxidase. This study further characterized the Mn(II)-oxidizing activity and MG decolorization capability of StKatG2. Additionally, the efficient heterologous expression of StKatG2 enhanced its potential utility in environmental remediation.

## 2 Materials and methods

### 2.1 Phylogenetic analysis and structural alignment

The catalase-peroxidase sequences were obtained from PeroxiBase^1^ (Passardi et al., 2007), where they have been verified and annotated. Multiple sequence alignment was performed using Clustal Omega^2^, while structural alignment was conducted with the ESPript program suite^3^. The phylogenetic tree was generated using MEGA7, employing the maximum likelihood method as described by Kumar et al. (2016) and the Jones-Taylor-Thornton (JTT) model, with 1,000 bootstrap replicates (Jones et al., 1992). The structure of StKatG1 and StKatG2 was predicted using the machine learning algorithm AlphaFold3 (v3.0)^4^. PyMOL software^5^ was utilized for the structural alignment of the homology model with the AlphaFold3 structure, as well as for calculating and visualizing root mean square deviations.

### 2.2 Cloning of *stkatG2* gene from *Salinicola tamaricis* F01

The *Salinicola tamaricis* F01 strain was cultured on LB5 medium (peptone 10 g/L, yeast powder 5 g/L, NaCl 50 g/L, agar 15 g/L) at 30°C for 48 h. Genomic DNA of strain F01 was isolated using the Qiagen genomic DNA isolation kit. The *stkatG2* gene was amplified from the genomic DNA using the primers 5’-GGAATTCCATATGATGAGT GAAGAGATCAAGATCGGTGG-3′ (with the *Nde*I restriction site underlined) and 5’-CGCGGATCCGAGACGTCGAAGCGGTC GTG-3′ (with the *Bam*HI restriction site underlined) for cloning into the pET-22b (+) expression vector, which includes a C-terminal hexahistidine tag. The recombinant plasmid was constructed as described and subsequently transformed into *Escherichia coli* BL21 competent cells (Vazyme, Nanjing, China). Transformants were selected on LB24 medium (peptone 10 g/L, yeast powder 24 g/L, NaCl 50 g/L, agar 15 g/L) with 50 μg/mL ampicillin.

### 2.3 Expression and purification of recombinant StKatG2

The correct transformants were cultured to an OD_600 nm_ of 0.6 in LB24 medium containing 50 μg/mL ampicillin. To induce the expression of recombinant StKatG2, 0.05 mmol/L isopropyl-*β*-d-thiogalactopyranoside (IPTG) was added. The culture was incubated at 20°C for 10 h. Cells were lysed by suspending them in a 50 mmol/L solution of Tris–HCl (pH 7.4) and sonicated on ice to facilitate further disruption (20 kHz power for 12 min with 5 s burst sonication cycles at 5 s intervals). At 4°C, the crude enzyme solution was purified using a nickel column (Yeasen, Shanghai, China), with 4 mL of HisSep Ni-NTP Agarose Resin loaded into the purification column. Lysis Buffer (50 mmol/L NaH_2_PO_4_, 300 mmol/L NaCl, 10 mmol/L imidazole, pH 8.0) was employed for equilibration, followed by the introduction of 20 mL of crude enzyme solution into the nickel column resin. After washing away impurities with Wash Buffer (50 mmol/L NaH_2_PO_4_, 300 mmol/L NaCl, 20 mmol/L imidazole, pH 8.0), StKatG2 was eluted using Elution Buffer (50 mmol/L NaH_2_PO_4_, 300 mmol/L NaCl, 250 mmol/L imidazole, pH 8.0) and 7.5 mL was collected, and subsequently dialyzed for 12 h in a 50 mmol/L Tris–HCl solution (pH 7.4). The purified protein was analyzed by SDS-PAGE, and the StKatG2 protein concentration was determined using a BCA kit (Yeasen, Shanghai, China).

To evaluate the molecular weight of the proteins, purified protein samples were subjected to chromatographic separation and molecular weight determination using High-Performance Liquid Chromatography-Mass spectrometry (HPLC-MS) technology (Thermo Fisher Scientific, United States, Ultimate 3,000 Ultra High Performance Liquid Chromatography System, Q Exactiv™ Hybrid Quadrupole-Orbitrap™ Mass Spectrometer High Resolution Mass Spectrometer).

### 2.4 Mn(II)-oxidizing activity analysis

Manganese chloride (MnCl_2_) was employed as the substrate to evaluate the Mn(II) oxidation activity of the recombinant StKatG2 enzyme in HEPES buffer at pH 8.0. The buffer composition included 59 mmol/L NaCl, 10 mmol/L CaCl_2_, 0.4 mmol/L NADḤNa_2_ (Sigma), 1  mmol/L H_2_O_2_, 1 μmol/L heme (Sigma) in 50 mmol/L, and 10 μmol/L pyrroloquinoline quinone (Sigma) (Kono and Fridovich, 1983). The reaction mixture was incubated for 10 h at 30°C. A 300 μL aliquot of each sample was reacted for 2 h at 30°C with 60 μL of LBB (Sigma, 0.04%, w/v) (Jones et al., 2019) and 900 μL of acetic acid (45 mmol/L). The activity was assessed by monitoring the absorbance at 620 nm and the equilibrated potassium permanganate concentration.

To determine the kinetic constant of recombinant StKatG2, various concentrations of MnCl_2_ (ranging from 0–80 mmol/L) were incubated with 0.4 mg/mL of StKatG2 for 10 h at 30°C in 50 mmol/L Tris–HCl (pH 7.4) (Jang et al., 2005). The values for *K*_m_ and *V*_max_ were derived using non-linear regression fitting.

### 2.5 Impact of temperature, pH, and metal ions

The effect of temperature on the enzymatic activity of StKatG2 was investigated by incubating the reaction mixture at temperatures ranging from 25 to 80°C under standard assay conditions at pH 7.0. The residual enzyme activity was evaluated at 50°C, with the maximum enzyme activity defined as 100%. Additionally, the optimal pH was determined by varying pH values from 4.0 to 8.0 while maintaining a constant temperature of 50°C.

To assess thermostability, StKatG2 samples were incubated at different temperatures (50, 60, 70, and 80°C) for various time intervals. Subsequently, residual activity was measured at 50°C and compared with protein samples stored on ice, which was considered to represent 100% activity.

EDTA and several metal ions, including Ca^2+^, Co^2+^, Cu^2+^, Fe^3+^, Mg^2+^, Zn^2+^, and Ni^2+^, were incubated with StKatG2 at final concentrations ranging from 0.1 mmol/L to 10 mmol/L at 50°C for a duration of 10 h. The effects of these ions on the activity of recombinant StKatG2 were then evaluated.

### 2.6 Characterization of BioMnOx

The MnCl_2_ (50 mmol/L) and various auxiliary components were reacted with recombinant StKatG2 (0.5 mg/mL) at 55°C for 10 h. The fresh Mn oxides (BioMnOx) pellets were collected by centrifugation (10,000 × *g*, 10 min), and washed three times with deionized water before drying for 12 h at 55°C. Surface topography observations were performed using a scanning electron microscope (Thermo Fisher Scientific, United States, Apreo 2C) at 5.00 kV, equipped with an Oxford X-Max 80T EDX system (Oxford Instruments Ultim Max 65, Oxford, United Kingdom) for energy dispersive analysis of BioMnOx.

The phases were determined by scanning the dry form of the dioxide using an X-ray diffractometer (Beijing Purkinje General Instrument Co, China, X-D6) in the 2θ range of 5–85°. The valence state and composition of Mn were identified using the X-ray photoelectron spectroscopy (XPS; ESCALAB XI+, Thermo Fischer, United States).

### 2.7 Decolorization of MG

The MG decolorization experiments were conducted in 2 mL tubes using a reciprocating shaker set at 30°C and 120 rpm. The reaction mixture comprised a 50 mmol/L Tris–HCl buffer containing 20 mg/L, 50 mg/L, and 80 mg/L of MG, along with 500 μg/mL of purified recombinant StKatG1 or StKatG2. Control groups were established that excluded the StKatG1 or StKatG2 enzymes. The absorbance of MG at 620 nm was measured using a UV spectrophotometer. The formula for calculating the decolorization rate is as follows:


$$\begin{array}{l} \mathrm{Decolorization}\left(\%\right)=\\ \frac{\mathrm{initial}\;MG\;\mathrm{absorbance}-\mathrm{final}\;MG\;\mathrm{absorbance}}{\mathrm{initial}\;MG\;\mathrm{absorbance}}*100\% \end{array}$$

All experiments were performed in triplicate.

### 2.8 HPLC-MS analyses of MG metabolites

High-Performance Liquid Chromatography-Mass Spectrometry (HPLC-MS) was employed to detect malachite green (MG) and its degradation products (Daneshvar et al., 2007). The malachite green or its metabolites were dried, dissolved in 1 mL of methanol, and subsequently injected into an Agilent 1,290 Infinity II HPLC system (Agilent, Santa Clara, United States) at a volume of 3 μL. This system was equipped with a reversed-phase C-18 analytical column and coupled to a quadrupole mass spectrometer. Methanol served as the mobile phase, with a flow rate of 0.6 mL min^−1^. The initial methanol proportion was set at 5%, which was gradually increased to 95% over 10 min, maintained at 95% for an additional 2 min, and then reduced back to 5% over 2 min. The entire analytical process lasted for a total of 20 min (Han et al., 2020). The analysis utilized electrospray ionization (ESI) in positive ion mode, and the data were processed using Qualitative Analysis B.05.00 software (Agilent, United States).

### 2.9 Molecular docking studies

The protonation state of all compounds was set at pH 7.4, and the compounds were converted to three-dimensional structures using Open Babel (O'Boyle et al., 2011). AutoDock Tools (ADT3) were utilized to prepare and parametrize the receptor protein and ligands. Docking grid files were generated using the AutoGrid feature of Sitemap, and docking simulations were performed with AutoDock Vina (1.2.0) (Trott and Olson, 2010; Eberhardt et al., 2021). The optimal pose was selected for interaction analysis. Finally, the protein-ligand interaction figure was generated using PyMOL.

## 3 Results

### 3.1 Sequence and structural differences between StKatG1 and StKatG2 proteins

**Figure 1:**
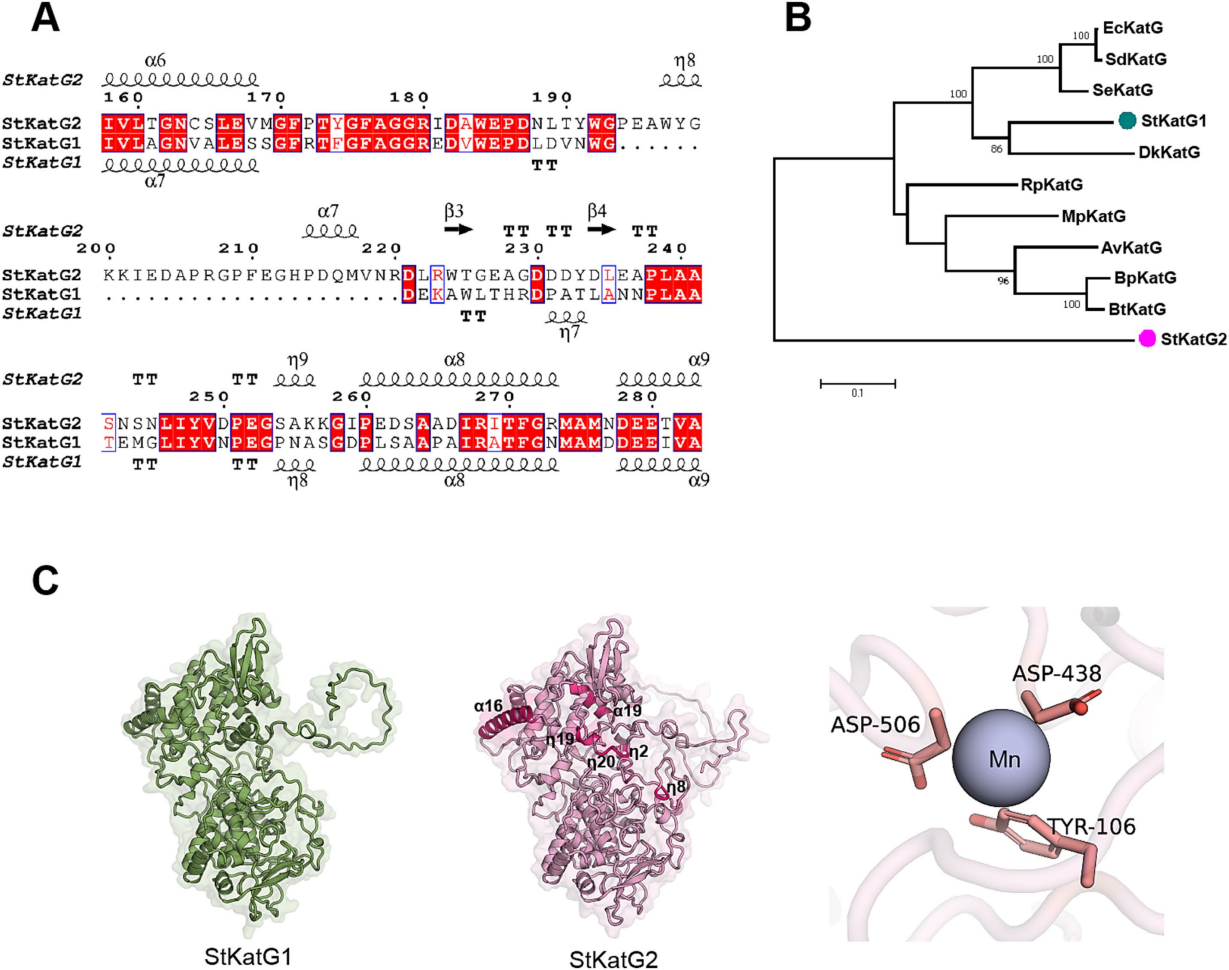
Comparison of amino acid sequences of StKatG homologs. (A) Sequence alignment of StKatG1 and StKatG2. The sequences were aligned using the Clustal Omega and treated with ESPript 3 (https://espript.ibcp.fr/). The secondary structure elements presented on top were obtained from the predicted StKatG2 structure using Alphafold3 (α, α-helices; *η*, 3 10 helices; *β*, β-strands. TT, turns). Identical and similar residues are displayed in red and blue boxes, respectively. (B) Phylogenetic tree of KatG proteins from *Salinicola tamaricis* and other species. StKatG1 and StKatG2, *Salinicola tamaricis* KatGs; DkKatG, *Drosophila kikkawai* KatG; BpKatG, *Burkholderia pseudomallei* KatG; SeKatG, *Salmonella enterica* KatG; EcKatG, *Escherichia coli* KatG; SdKatG, *Shigella dysenteriae* KatG; RpKatG, *Ralstonia pickettii* KatG; BtKatG, *Burkholderia thailandensis* KatG; MpKatG, *Methylibium petroleiphilum* KatG; AvCP01, *Azotobacter vinelandii* KatG. The evolutionary analyses were conducted based on amino acid homology using the MEGA7. Bootstrap values are shown at branch points. (C) Predicted structures of StKatG1 and StKatG2 using AlphaFold3, with the manganese binding site in StKatG2 represented as a purple sphere.

Two catalase-peroxidases, StKatG1 (~80 kDa) and StKatG2 (~85 kDa), were identified from *S. tamaricis* F01. While StKatG1 has been confirmed to exhibit Mn(II) oxidation activities in previous research (Zhao et al., 2017; Zhao et al., 2023), the function of StKatG2 remains uncharacterized. StKatG2 shares 53.6% amino acid sequence identity with StKatG1 (Figure 1A and Supplementary Figure S1). Phylogenetic analysis revealed that StKatG1 clusters with catalase-peroxidases from *Burkholderia pseudomallei*, *Salmonella enterica*, and *Escherichia coli*, among others, whereas StKatG2 is distantly related (Figure 1B). Alphafold3 (v3.0) analysis indicated that StKatG2 possesses a typical catalase-peroxidase structure, comprising 23 *α*-helices, 22 short 3 10 helices, and 13 *β*-strands, with the critical histidine-arginine pair (His-112 and Arg-108) located in α-helix 4, essential for H_2_O_2_ breakdown (Poulos and Kraut, 1980). Additionally, StKatG2 also contains the triad His295-Asp406-Trp347 as the proximal heme iron ligand, along with Asp-130, Arg-444, and Tyr-248, which act as a molecular switch regulating catalase activity (Regelsberger et al., 2000; Jakopitsch et al., 2003; Yu et al., 2003; Singh et al., 2004; Carpena et al., 2005). A comparison of the structures of StKatG1 and StKatG2 revealed significant similarities, with a root mean square deviation (RMSD) of 0.98 Å. Both StKatG1 and StKatG2 exhibit three types of secondary structures: *α*-helices, short 3_10 helices, and β-strands. StKatG1 contains 24 α-helices, whereas StKatG2 has 23. In contrast, StKatG1 features 17 3_10 helices, while StKatG2 possesses 22. Regarding *β*-strands, StKatG1 has 10, compared to 13 in StKatG2 (Figure 1C). In conclusion, despite being derived from the same organism, StKatG1 and StKatG2 demonstrate notable distinctions in both sequence and structure.

### 3.2 Expression and purification of recombinant StKatG2

To investigate the function of the StKatG2 protein, the full-length gene (2,297 bp) was cloned and inserted into the pET-22b (+) expression vector with a His6-tag at the C-terminal (Supplementary Figures S2, S3). The resulting recombinant plasmid was confirmed through double digestion with *Nde*I and *Bam*HI restriction enzymes, yielding a correct 2.3 kb DNA fragment. Subsequently, the recombinant plasmid was transformed into *E. coli* BL21, and transformants on LB medium supplemented with ampicillin (50 μg/mL) were selected via colony PCR. The purified recombinant StKatG2 protein was obtained using an immobilized Ni^2+^-affinity column. A total of 20 mL of crude proteins (1792.71 μg/mL) was subjected to an immobilized Ni^2+^-affinity column, resulting in 7.5 mL of eluted proteins with a concentration of 229 μg/mL. Analysis by SDS-PAGE (10%) revealed a band at approximately about 85 kDa (Supplementary Figures S4, S5), consistent with the expected mass of StKatG2. The HPLC-MS result demonstrated that the actual molecular weight of StKatG2 was 86.97 kDa (Supplementary Figure S6B and Supplementary Tables S1, S2).

### 3.3 Characterization of the manganese-oxidizing activity of StKatG2

**Figure 2:**
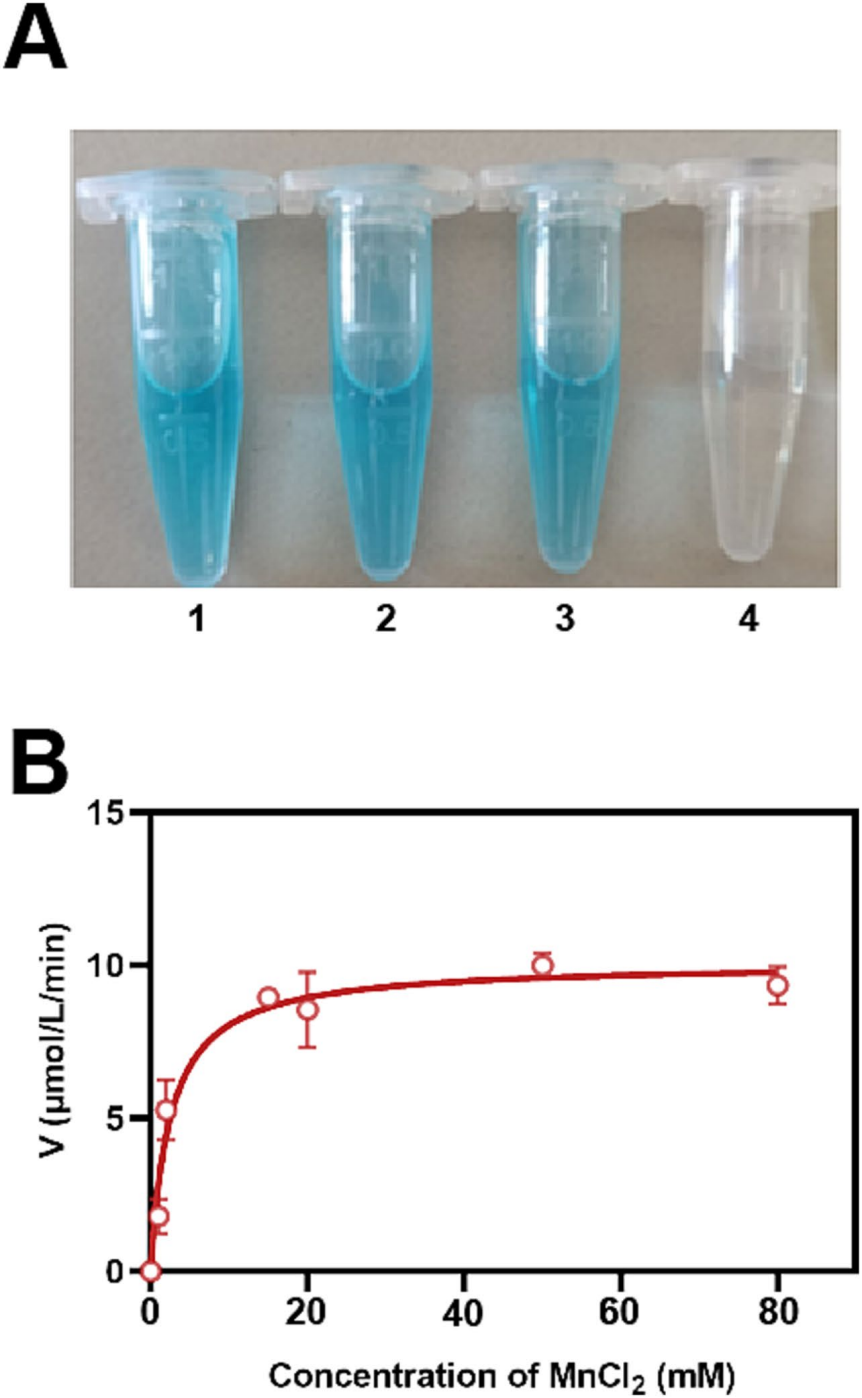
Characterization of StKatG2. (A) The LBB assay for the formation of Mn oxides by incubating purified recombinant StKatG2 (0.3 mg protein/mL) with MnCl_2_ (50 mmol/L). Tube 1–3: sample after 24 h of incubation in the presence of StKatG2; tube 4: Negative control after 24 h in the absence of StKatG2. (B) The reaction kinetics of StKatG2 (0.3 mg protein/mL) were analyzed using various concentrations of MnCl_2_. The data presented are the averages of three independent experiments, accompanied by standard error bars. The curve was fitted using the Michaelis–Menten equation.

The manganese-oxidizing activity of recombinant StKatG2 was assessed using the LBB method (Jones et al., 2019). Upon reaction of StKatG2 with MnCl_2_ in 50 mmol/L Tris–HCl (pH 7.4) buffer, insoluble brown particles were observed. Subsequent mixing of the reaction solution with the LBB solution resulted in a blue color change (Figure 2A). These reactions confirmed the formation of Mn oxides, demonstrating the ability of recombinant StKatG2 to oxidize Mn(II).

The oxidation ability of StKatG2 toward Mn(II) was further analyzed by calculating kinetic values at different concentrations of MnCl_2_. A Michaelis–Menten plot was generated through non-linear regression curve fitting (Figure 2B). Table 1 provides a comparison of the kinetic characteristics of various manganese-catalases. It is evident that the *K*_m_ value of StKatG2 is 2.529 mmol/L, higher than that of StKatG1 (Zhao et al., 2023). Moreover, the *K*_m_ value of StKatG2 was found to be lower than those of most reported multicopper oxidases, suggesting that StKatG2 has a higher affinity for the substrate and greater manganese oxidation capacity. The *V*_max_ value for StKatG2 was determined to be 10.07 μmol/L·min^−1^, slightly lower than StKatG1 (Zhao et al., 2023), which exhibited a *V*_max_ of 10.3 μmol/L·min^−1^. This implies that StKatG1 has a slightly higher maximum velocity in the catalytic reaction compared to StKatG2. The *K*_cat_ of StKatG2 is 2.82 min^−1^, slightly higher than that of StKatG1 with a *K*_cat_ value of 2.78 min^−1^, indicating a slightly superior catalytic performance in terms of substrate turnover for StKatG2. These differences in kinetic values between StKatG1 and StKatG2 may be attributed to variances in enzyme structure, active site residues, or the microenvironment surrounding the active site, which could impact the efficiency of the catalytic reaction.

**Table 1:** Comparison of the kinetic parameters for StKatG2 and other characterized manganese oxidases.

Name	*K*_m_	*V*_max_	*K*_cat_	Reference
CueO	17.33 mM	9.33 μM min^−1^	2.09 s^−1^	Su et al. (2014)
CotA	14.85 mM	3.01 μM min^−1^	0.32 s^−1^	Su et al. (2013)
MopA	154 μM	1.08 μM min^−1^	1.60 min^−1^	Medina et al. (2018)
StKatG1	1.29 mM	10.3 μM min^−1^	2.78 min^−1^	Zhao et al. (2023)
StKatG2	2.529 mM	10.07 μM min^−1^	2.82 min^−1^	This study

### 3.4 Characterization of BioMnOx

**Figure 3:**
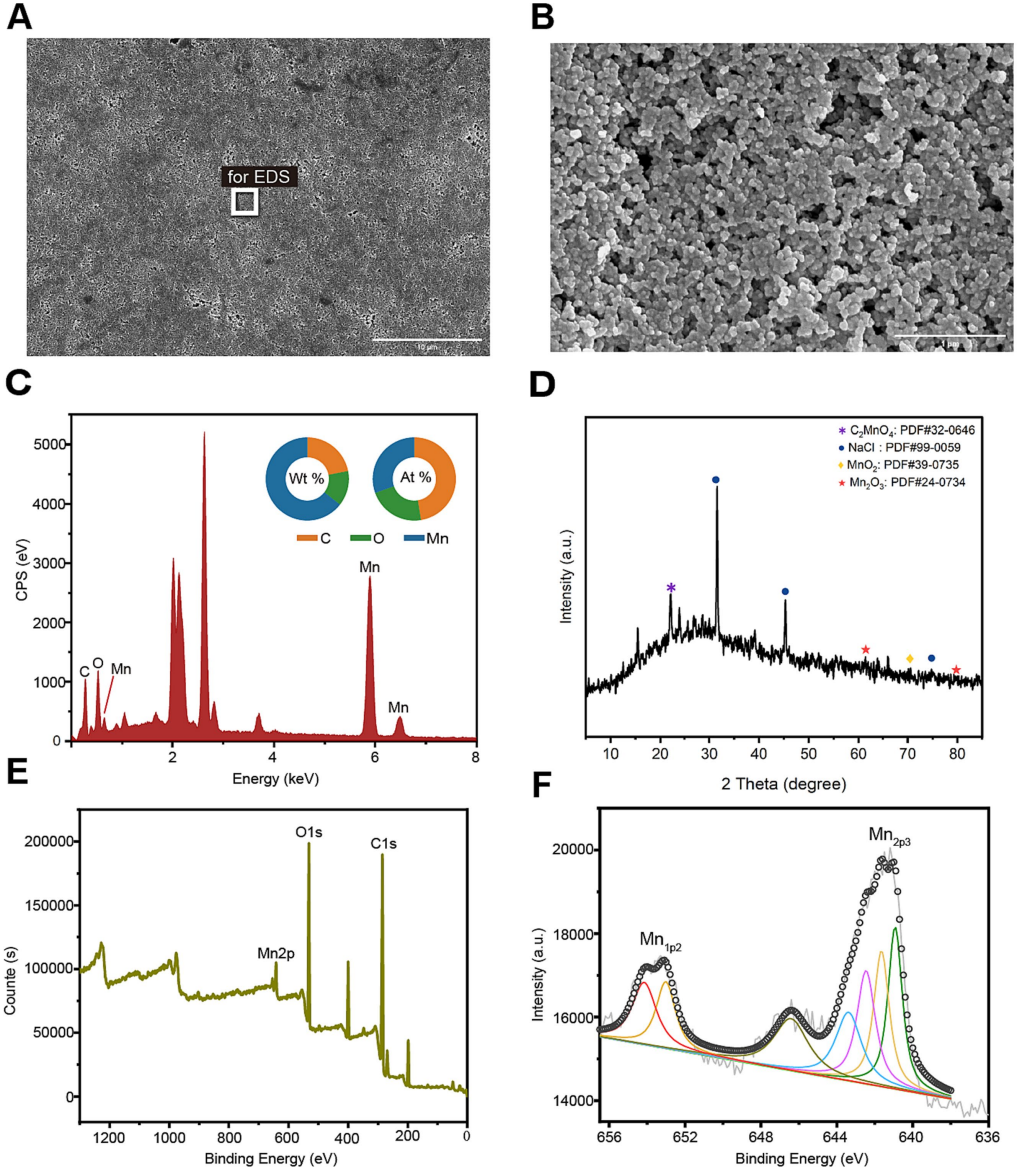
Morphology and composition of BioMnOx catalyzed by StKatG2. The BioMnOx were obtained through incubation of MnCl_2_ (50 mmol/L) and StKatG2 (0.5 mg/mL) at 50°C for 10 h. (A) The SEM photograph of the Mn oxide aggregates produced by StKatG2. Scale bar, 10 μm. (B) The SEM photograph of the Mn oxide aggregates at a different magnification with (A). Scale bar, 1 μm. (C) The EDS spectrum showing the Mn composition of the aggregates. The rectangle shown in figure A indicates the selected position of the aggregates. The unlabeled peaks are Au formed by the spray gold treatment. (D) The XRD pattern of the BioMnOx produced by StKatG2. (E) XPS wide scan patterns. (F) The deconvoluted profile of the specific Mn 2p_1/2_ and Mn 2p_2/3_ spectrum for the BioMnOx.

In order to further characterize the biogenic manganese oxides (BioMnOx) catalyzed by StKatG2, scanning electron microscopy (SEM) combined with energy dispersive spectroscopy (EDS) was conducted. Unlike layered sheet-like structure of BioMnOx catalyzed by StKatG1 (Zhao et al., 2023), the BioMnOx nanoparticles produced by StKatG2 appeared somewhat spherical or irregular, arranged closely with void spaces between them (Figures 3A,B). EDS analysis revealed the elemental composition on the surface of the BioMnOx particles, with mass fractions of 21.97% C, 13.64% O, and 64.39% Mn, and atomic fractions of 47.46% C, 22.12% O, and 30.41% Mn (Figure 3C).

To analyze the phase of the product, X-ray diffraction (XRD) was utilized for qualitative analysis of the phase composition of bio-manganese oxide (Figure 3D). The results revealed that the primary phase composition of bio-manganese oxide consisted of MnO_2_ (JCPDS 39–0735), Mn_2_O_3_ (JCPDS 24–0734), and C_2_MnO_4_ (JCPDS 32–0646). These findings suggest that the Mn compounds catalyzed by StKatG2 exhibit multiple oxidation states. Furthermore, notable peaks of NaCl (JCPDS 99–0059) were observed, likely attributed to the incorporation of sodium chloride during the preparation of BioMnOx.

Valence states compositions in the BioMnOx were identified through photoelectron spectroscopy (XPS). The XPS analysis showed two peaks in Mn2p_1/2_ and five in Mn2p_3/2_ (Figure 3E), consistently indicating that the BioMnOx produced by StKatG2 is a mixed-valence manganese compound. Specifically, XPS measurements revealed the presence of four valence states: Mn(II), Mn(III), Mn(IV), and Mn(VII) in the BioMnOx (Figure 3F). The multiple oxidation states in the BioMnOx product catalyzed by StKatG2 are similar to those catalyzed by StKatG1, which also exhibits mixed valences (Zhao et al., 2023).

### 3.5 The effect of pH and temperature on the Mn(II)-oxidizing activity of StKatG2

**Figure 4:**
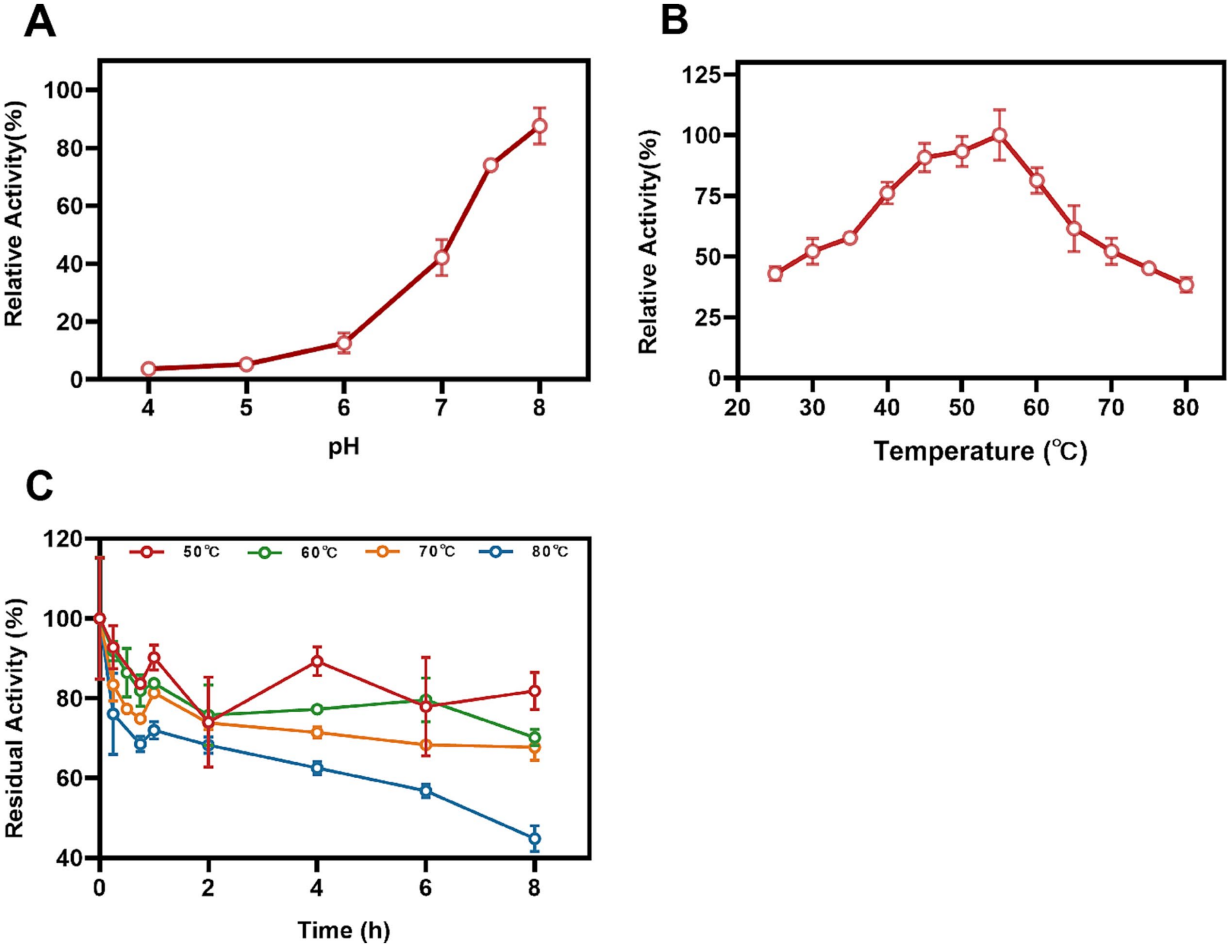
Effects of temperature and pH on Mn(II)-oxidizing activities of StKatG2. (A) Mn(II)-oxidizing activities of StKatG2 at different pH values. (B) Mn(II)-oxidizing activities of StKatG2 at different temperatures. (C) Thermostability of StKatG2 at various temperatures. StKatG2 was incubated at 50°C (red circles), 60°C (green circles), 70°C (orange triangles), and 80°C (blue triangles) for different time intervals, with residual activities measured at 50°C. The error bars indicate standard deviation.

The impact of pH conditions on the Mn(II)-oxidizing activity of StKatG2 was investigated. The optimal pH for StKatG2 to exhibit Mn(II)-oxidizing activity was found to be 7.5 (Figure 4A). The relative enzyme activity of StKatG2 is only 8.3% at pH 4.0, and the highest enzyme activity was observed around 7.5, which indicated that neutral or slightly alkaline environments were beneficial to the Mn(II)-oxidizing activity of StKatG2. This trend is consistent with other studies on manganese oxidases, such as CopA with an optimal pH of 8.0. CueO, another manganese oxidase, shows peak activity between pH 7.6 and 8.2, with minimal oxidation activity below pH 7.0 (Su et al., 2014). These findings suggest that most manganese oxidases involved in biomanganese oxidation exhibit maximal enzyme activity in neutral to alkaline pH ranges, while chemical oxidation of manganese becomes predominant at pH levels above 8.0, as demonstrated by the control sample without StKatG2 (Supplementary Figure S7).

To determine the optimal temperature for Mn(II) oxidizing activity, StKatG2 was evaluated across a temperature range of 25–80°C. The study revealed that the recombinant StKatG2 exhibited its peak enzymatic activity at 55°C (Figure 4B). Notably, the enzyme retained 40% activity at lower temperatures (25°C) and maintained over 80% activity within the 45–55°C range. However, enzyme activity significantly decreased beyond 55°C, with a sharp drop to 38.3% at 80°C. These findings indicate that StKatG2 is a mesophilic enzyme. Moreover, the optimal temperature for manganese oxidase activity in recombinant StKatG2 was higher than that observed for StKatG1 (50°C) (Zhao et al., 2023).

Thermostability of StKatG2 was evaluated by subjecting the protein to different temperatures (50, 60, 70, and 80°C) for a specific duration, followed by measuring residual activity at 55°C (Figure 4C). After an 8-h exposure to 50°C, the enzyme retained 82.3% of its activity, surpassing StKatG1, which only maintained 73.6% activity under similar conditions (Zhao et al., 2023). Notably, StKatG2 demonstrated superior thermal stability, retaining 45.1% activity after 8-h exposure to 80°C. In contrast, StKatG1 lost 92% of its activity when exposed to 80°C for 8 h (Zhao et al., 2023). These results collectively indicate that StKatG2 exhibits robust thermal stability in high-temperature environments and shows promise for broader applications in such environments.

### 3.6 The effect of metal ions on the enzymatic activity of StKatG2

**Figure 5:**
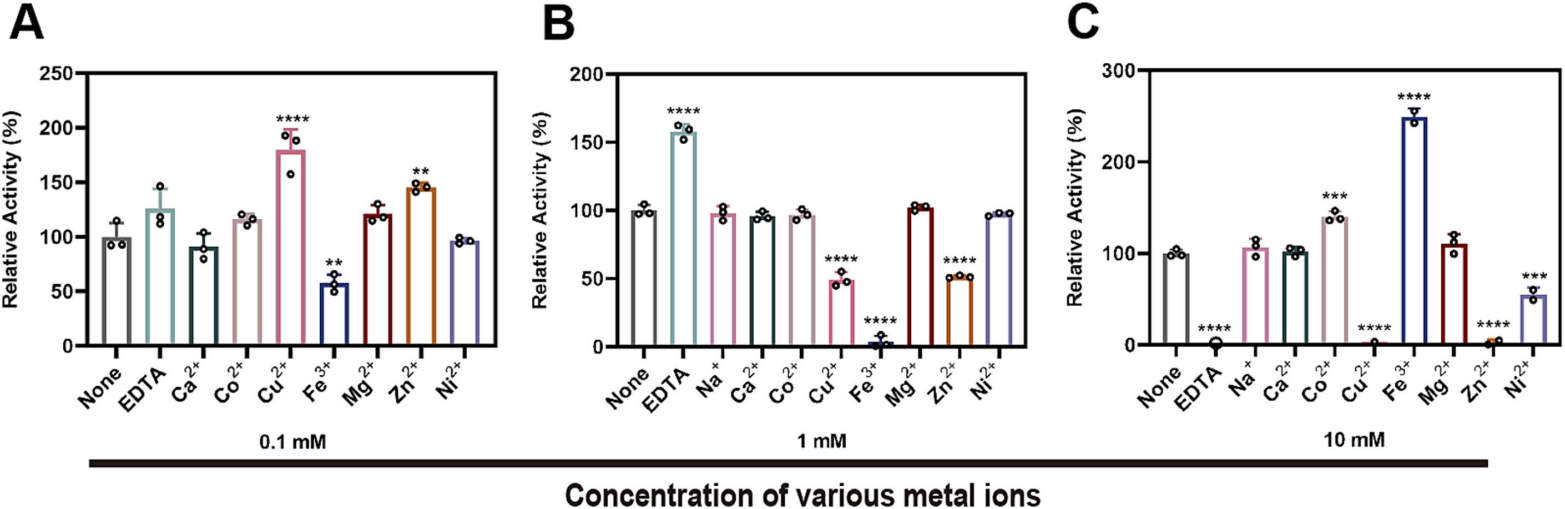
Effects of different metal ions and EDTA on the Mn(II)-oxidizing activity of StKatG2. Effects of different concentrations of metal ions and EDTA at 0.1 mM (A), 1 mM (B), and 10 mM (C) on enzymatic activity the Mn(II)-the oxidizing activity of StKatG2. **, ***, and **** represent significant differences from the control group (**p < 0.01, ***p < 0.001, ****p < 0.0001).

The impact of metal ions and the chelating agent EDTA on the enzymatic activity of StKatG2 was assessed (Figure 5). EDTA significantly suppressed the Mn(II)-oxidizing activity of StKatG2, suggesting a reliance on divalent metal ions. At a concentration of 0.1 mmol/L, Fe^3+^ inhibited the enzyme activity (Figure 5A), likely because of its structural and chemical similarity to Mn, leading to potential competition for binding sites on the enzyme and consequent disruption of its structure. Moreover, the addition of 0.1 mmol/L Cu^2+^, Mg^2+^, and Zn^2+^ enhanced the enzyme activity, possibly by forming a metalloenzyme complex that boosts enzymatic function. However, a higher concentration (10 mmol/L) of Cu^2+^ and Zn^2+^ almost completely inhibited the enzymatic activity, while Fe^3+^ at the same concentration affected the final detection outcome due to its inherent color, which holds no analytical value (Figure 5C).

### 3.7 Decolorization ability of recombinant StKatG2 on MG

**Figure 6:**
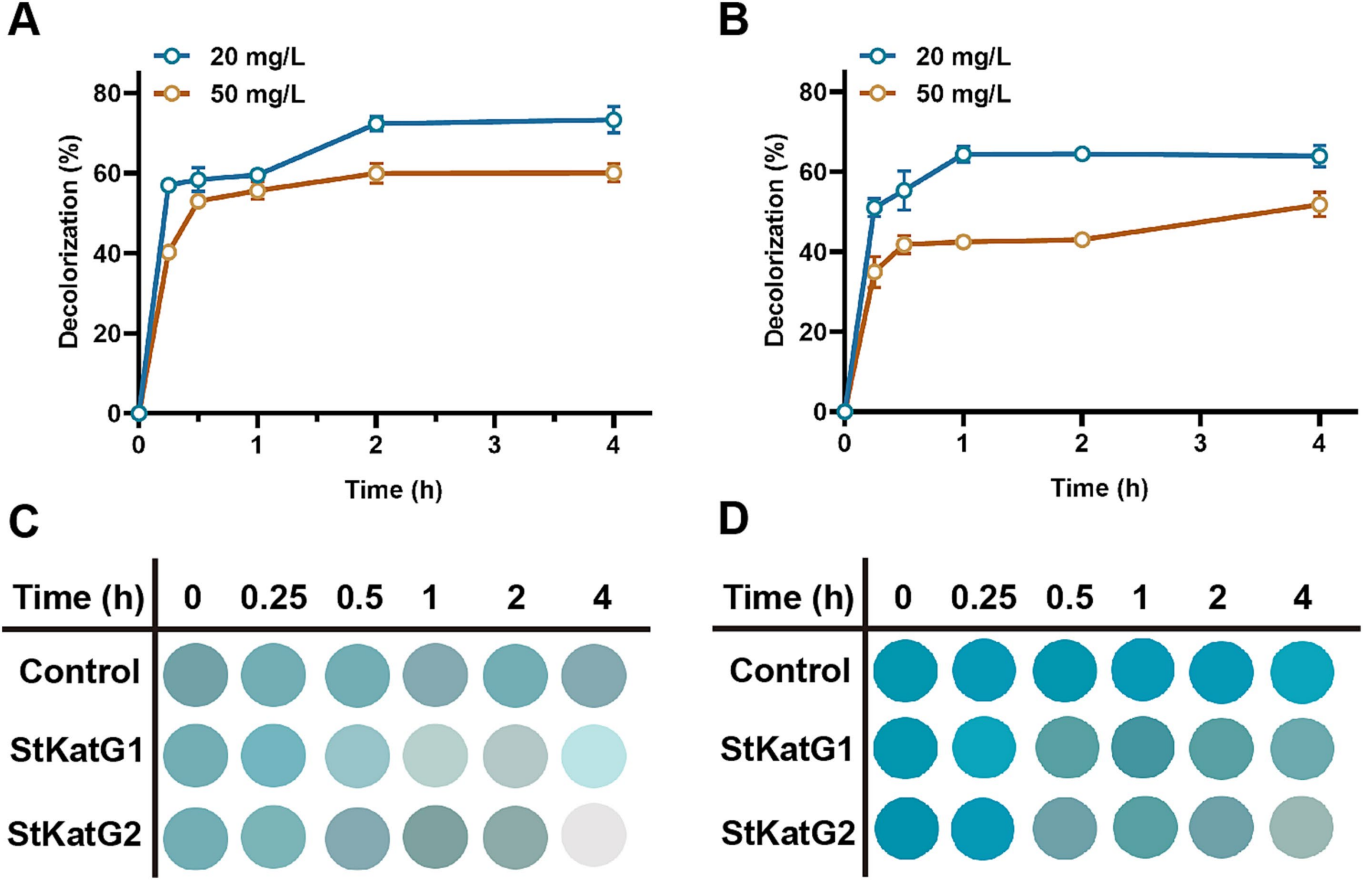
Decolorization of MG mediated by StKatGs. (A) The decolorization rate of MG by StKatG2. (B) The decolorization rate of MG by StKatG1. (C) Decolorization of MG at an initial concentration of 20 mg/L by StKatG2 and StKatG1. (D) Decolorization of MG at an initial concentration of 50 mg/L by StKatG2 and StKatG1.

To evaluate the performance of recombinant StKatG2, toxic malachite green was utilized as the research material. Upon mixing with the enzyme solution, the decolorization ability was monitored. StKatG2 exhibited the fastest decolorization efficiency within the initial 15 min for both 20 mg/L and 50 mg/L of malachite green, peaking around 30 min, with a decolorization rate of up to 30% after 4 h of action (Figure 6). Although the BioMnOx structures of StKatG1 and StKatG2 are similar and the kinetic values of Mn(II) are also comparable (Supplementary Table S3), StKatG2 exhibits excellent decolorization efficiency for malachite green. After 4 h of treatments, the maximum decolorization rates of StKatG2 reached 73.38% for 20 mg/L MG and 60.08% for 50 mg/L MG (Figure 6A), while StKatG1 for 20 mg/L mg and 50 mg/L mg were 63.91 and 51.78%, respectively (Figure 6B). When the concentration of MG reaches 80 mg/L, the decolorization rates of StKatG1 and StKatG2 were 34.63 and 18.37%, respectively (Supplementary Figure S8). These findings highlight StKatG2 as a promising biological approach for the efficient degradation of malachite green within a short period.

### 3.8 Mechanism of MG removal

**Figure 7:**
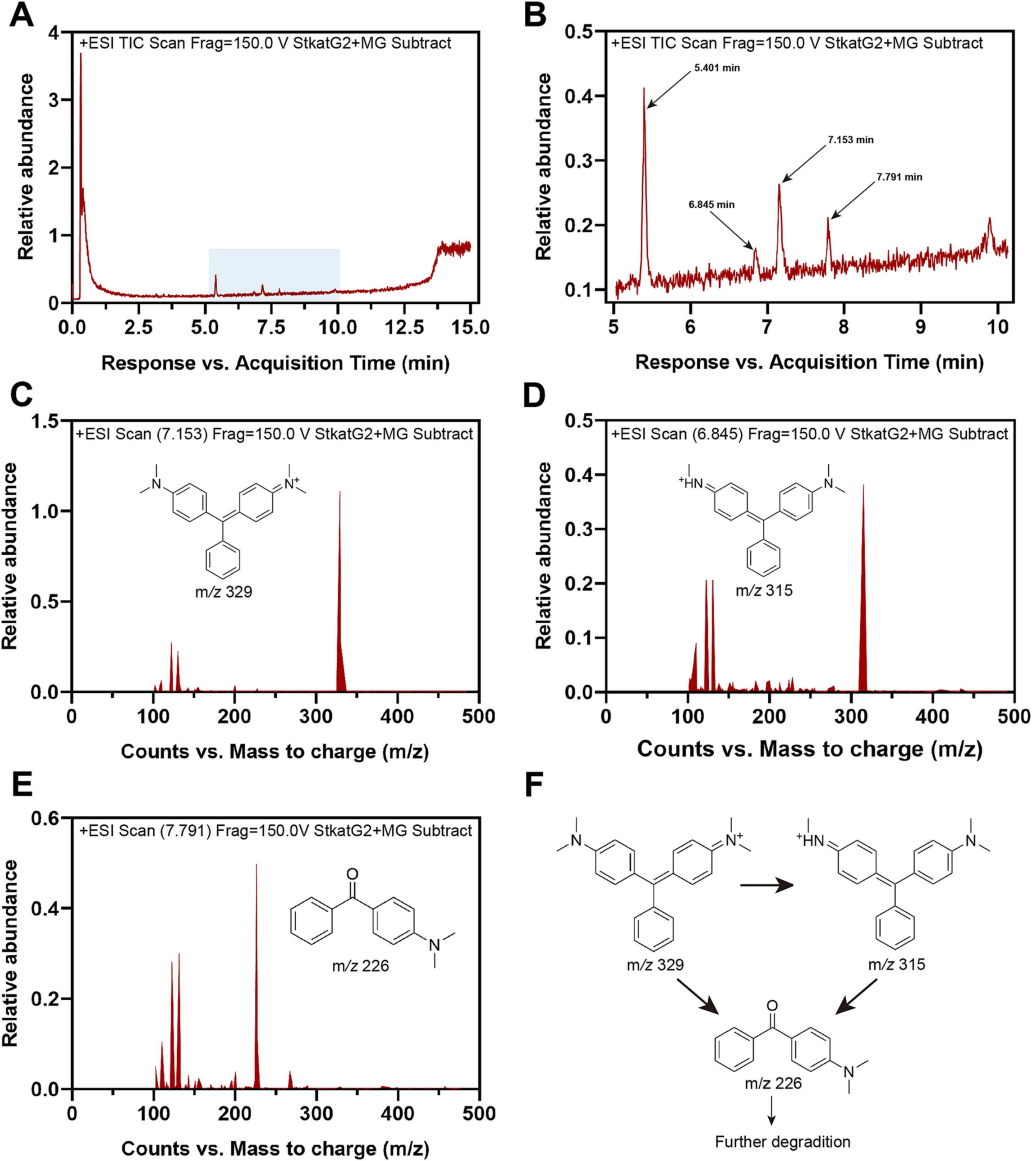
HPLC-MS analysis of MG metabolites catalyzed by StKatG2. (A) HPLC-MS spectrum of the MG degradation products by StKatG2. (B) Higher magnification view of the HPLC-MS spectrum of (A). (C) Malachite green (m/*z* = 329, retention time 7.153 min). (D) Leucomalachite Green (m*/z* = 315, retention time 6.845 min). (E) 4-(dimethylamino) benzophenone (m*/z* = 226, retention time 7.791 min). (F) Proposed removal pathways of MG by StKatG2, based on the results obtained from HPLC-MS.

To further investigate the decolorization mechanism of MG by treatment with StKatG2, the MG metabolites were analyzed using HPLC-MS. Following treatment with StKatG2, the MG peak, which exhibited a retention time of 6.845 min, was found to be reduced (Figure 7A). In contrast, several peaks with retention times ranging from 5 to 8 min increased (Figure 7B) when compared to the control setup. Three distinct peaks were identified as MG metabolites in the StKatG2 treatment setup (Figures 7C–E): malachite green (7.153 min, m/*z* = 329), leucomalachite green (6.845 min, m/*z* = 315), and 4-(dimethylamino) benzophenone (7.791 min, m/*z* = 226). The latter is recognized as a degradation metabolite of MG (Yang et al., 2015; Abu-Hussien et al., 2022). The process of MG degradation catalyzed by StKatG2 is illustrated in Figure 7F.

Furthermore, We conducted an analysis of the interactions between the proteins and MG (Supplementary Figure S9). Utilizing these interaction forces, the binding energy of the StKatG1-MG and StKatG2-MG complexes were determined to be −7.5 kcal/mol and −8.0 kcal/mol, respectively (Supplementary Figure S9). Molecular docking results indicate that MG interacts with both StKatG1 and StKatG2, with a position away from the heme binding sites, Mn^2+^ binding sites^,^ and the H_2_O_2_ catalytic pocket. Moreover, the docking positions of StKatG1 and StKatG2 with MG are inconsistent, which may contribute to the differing decolorization efficiencies observed for MG. When heme and Mn^2+^ combine with StKatG2 for manganese oxidation, this interaction may influence the molecular conformation of the proteins and release redox forces, ultimately leading to MG decolorization.

## 4 Discussion

The issue of textile industry dyeing wastewater is a significant concern in terms of wastewater pollution. Traditional dye restoration methods are often costly and can lead to the production of harmful byproducts. Enzymatic degradation technology offers a promising solution to these challenges, paving the way for a more sustainable environment. MG is a banned triphenylmethane dye that is still being used illegally in certain regions (Bañ Uelos et al., 2016; Zhou et al., 2019). Enzymes involved in the decolorization of triphenylmethane dyes can be categorized into two groups based on their specificity. The first group includes manganese peroxidases (Yang et al., 2016) and laccases (Casas et al., 2009), which act non-specifically through radical chain reactions to decolorize dyes (Azmi et al., 1998). The second group consists of enzymes specifically targeted at decolorizing triphenylmethane dyes, with triphenylmethane reductases being the sole members. However, these enzymes can only convert MG to LMG, which remains toxic to humans and other organisms (Persson et al., 2003; Zhou et al., 2019). In this study, we investigated the decolorization capabilities of MG using the catalase-peroxidases StKatG1 and StKatG2. With heme and methionine-tyrosine-tryptophan cofactors, catalase-peroxidase displays versatile functionality, exhibiting both peroxidase and catalase activities (Zámocký et al., 2009). Previous research has shown its effectiveness in treating wastewater containing Reactive Black 5, Allura Red, Fuchsine, and Acid Red 37 (Taslimi et al., 2013). Our findings further highlight the potential of catalase-peroxidase in decolorizing MG in textile bleaching effluent.

The catalase-peroxidase degradation pathway, while not completely understood, may play an indirect role in the degradation of dyes. Textile dyes can induce oxidative stress and generate reactive oxygen species (ROS). These ROS are then converted into hydrogen peroxide (H_2_O_2_) by superoxide dismutase (SOD), which is subsequently neutralized by catalase-peroxidase, leading to efficient dye removal under high-stress conditions (Takio et al., 2021). This study indicates that catalase-peroxidase StKatG2 also exhibits Mn(II)-oxidizing activities, which may synergistically enhance the removal of MG. This process is similar to that of another Mn(II) oxidase, MnP (Xiu, 2013). The heme in the enzyme reacts with H_2_O_2_ to generate highly reactive intermediates, causing the Mn(II) oxidase to transition into an oxidized state and facilitate the oxidation of Mn(II) to Mn(III). Subsequently, through a mechanism involving two successive electron transfer reactions, substrates like MG dyes can reduce the enzyme back to its original form. Mn(III) can be further oxidized to Mn(IV) via disproportionation (Soldatova et al., 2017; Tao et al., 2017), acting as an active oxidant and strong adsorbent, thereby enhancing the adsorption and oxidation of textile dyes (Zhou et al., 2018; Sun et al., 2021). Additionally, the BioMnOx produced by StKatG2 contains multiple valences, including Mn(II), Mn(III), Mn(IV), and Mn(VII). Mixed-valence Mn oxide generally exhibits higher electrical conductivity compared to single-valence states like Mn(III) or Mn(IV), making it more effective in adsorbing and oxidizing dyes (Zener, 1951). The synergistic effects of StKatG2’s multifunctionality may facilitate rapid start-up and stable operation in the removal of MG contaminants.

Comparatively, the catalase-peroxidase StKatG2 demonstrates a greater capacity to decolorize MG than StKatG1, despite their similar BioMnOx structures and comparable kinetic values for Mn(II). Although StKatG1 and StKatG2 share 53.6% amino acid identity, phylogenetic analysis indicates that they are distantly related. Notably, several differences exist between the two proteins, primarily located within the *α*-helix regions. Specifically, StKatG1 lacks the α7 helix found in StKatG2, while StKatG2 possesses a greater number of turns compared to StKatG1. This variation in the number of secondary structures may lead to deformation of the active pocket, resulting in differing binding efficiencies and varying decolorization effects.

Taken together, this study demonstrated that the catalase-peroxidase StKatG2 not only exhibited Mn(II)-oxidizing activity but also had the ability to decolorize MG. The successful cloning and overexpression of the StKatG2 gene in *E. coli* suggest a potential large-scale application for MG remediation in aquaculture. Furthermore, StKatG2 showed high stability at high temperatures and a wide range of pH and temperature tolerances, expanding its potential use in harsh environments. The findings of this research offer an environmentally-friendly solution that could be applied in aquaculture to effectively remove MG and reduce its accumulation in aquatic organisms, thereby ensuring the production of safe aquaculture products.

## Data Availability

The original contributions presented in the study are included in the article/Supplementary material, further inquiries can be directed to the corresponding author.

## Footnotes

^1^http://peroxibase.toulouse.inra.fr

^2^https://www.genome.jp/tools-bin/clustalw

^3^http://espript.ibcp.fr/ESPript/ESPript

^4^https://golgi.sandbox.google.com/

^5^http://www.pymol.org
