# Supplementary material for: Catalase-peroxidase StKatG2 from Salinicola tamaricis: a versatile Mn(II) oxidase that decolorizes malachite green
Source: Front Microbiol. 2024 Nov 5;15:1478305. doi: 10.3389/fmicb.2024.1478305 (PMC11573757; doi:10.3389/fmicb.2024.1478305)
Supplement: Supplementary file 1 [file Data_Sheet_1.PDF]

Supplementary data

Catalase-peroxidase StKatG2 from *Salinicola tamaricis*: a versatile Mn(II) oxidase that decolorizes malachite green

Mengyao Ding[1#], Wenjing Wang[1#], Zhenkun Lu[1], Yuhui Sun[1], Xinzhen Qiao[1], Meixue Dai[1], Guoyan Zhao[1*]

[1]College of Life Science, Shandong Normal University, Jinan 250014, China.

[#]These authors contributed equally: Mengyao Ding, Wenjing Wang.

*** Corresponding author:**

Guoyan Zhao, E-mail address: zhaoguoyan@sdnu.edu.cn, ORCID: 0000-0003-3200-8800

THIS SUPPLEMENTARY DOCUMENT CONTAINS:

NINE FIGURES AND THREE TABLES

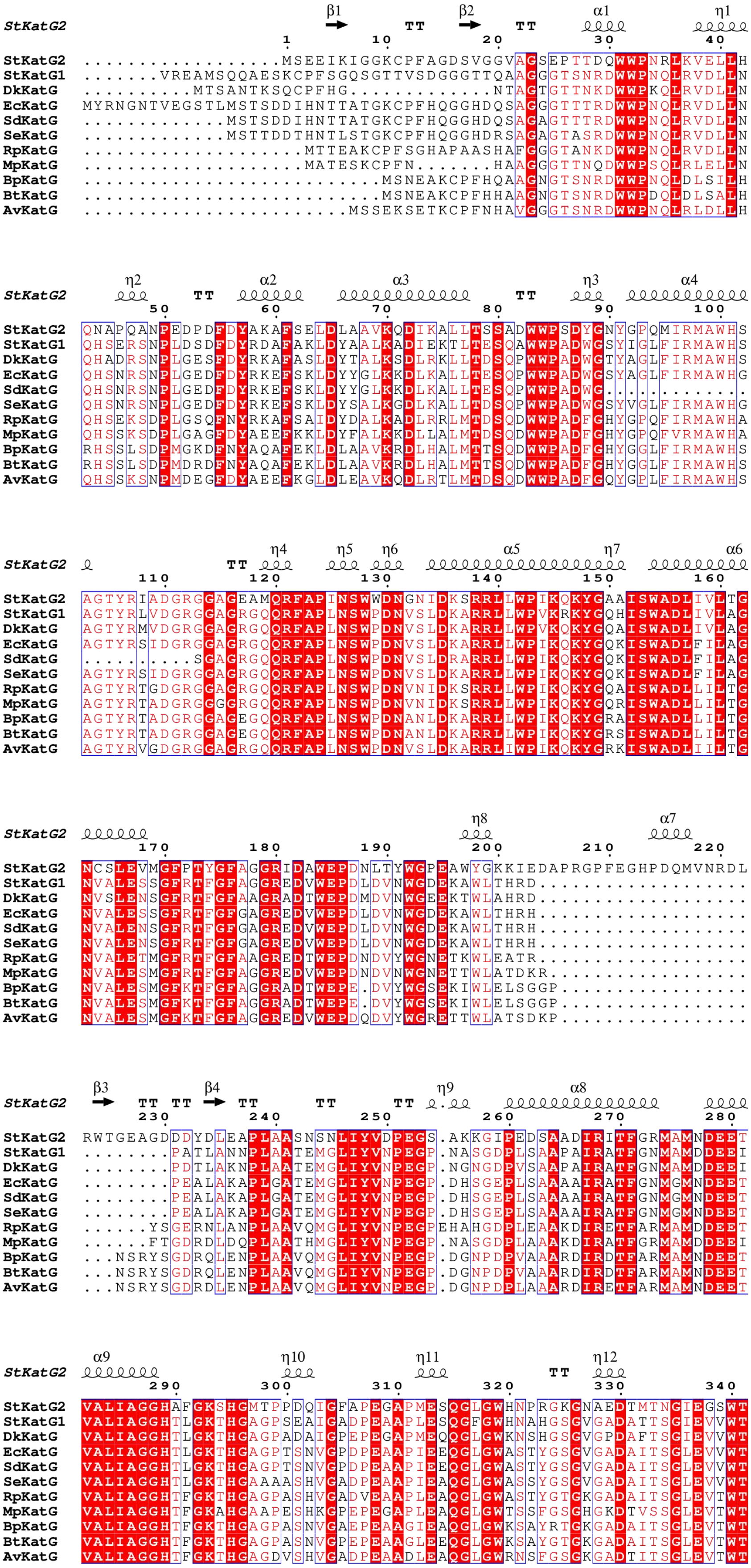

StKatG2
StKatG1
DkKatG
EcKatG
SdKatG
SeKatG
RpKatG
MpKatG
BpKatG
BtKatG
AvKatG
..................MSEEIKIGGKCPFAGDSVGGVAGSEPTTDQWWPNRLKVELLH
.......VREAMSQQAESKCPFSGQSGTTVSDGGGTTQAAGGGTSNRDWWPNQLRVDLLN
..........MTSANTKSQCPFHG............NTAGTGTTNKDWWPKQLRVDLLN
MYRNGNTVEGSTLMSTSDDIHNTTATGKCPFHQGGHDQSAGGGTTTRDWWPNQLRVDLLN
.............MSTSDDIHNTTATGKCPFHQGGHDQSAGAGTTTRDWWPNQLRVDLLN
.............MSTTDDTHNTLSTGKCPFHQGGHDRSAGAGTASRDWWPNQLRVDLLN
...................MTTEAKCPFSGHAPAASHAFGGGTANKDWWPNQLRVDLLN
.....................MATESKCPFN.......HAAGGGTTNQDWWPSQLRLELLN
.........................MSNEAKCPFHQAAGNGTSNRDWWPNQLDLSILH
.........................MSNEAKCPFHHAAGNGTSNRDWWPDQLDLSALH
......................MSSEKSETKCPFNHAVGGGTSNRDWWPNQLRLDLLH

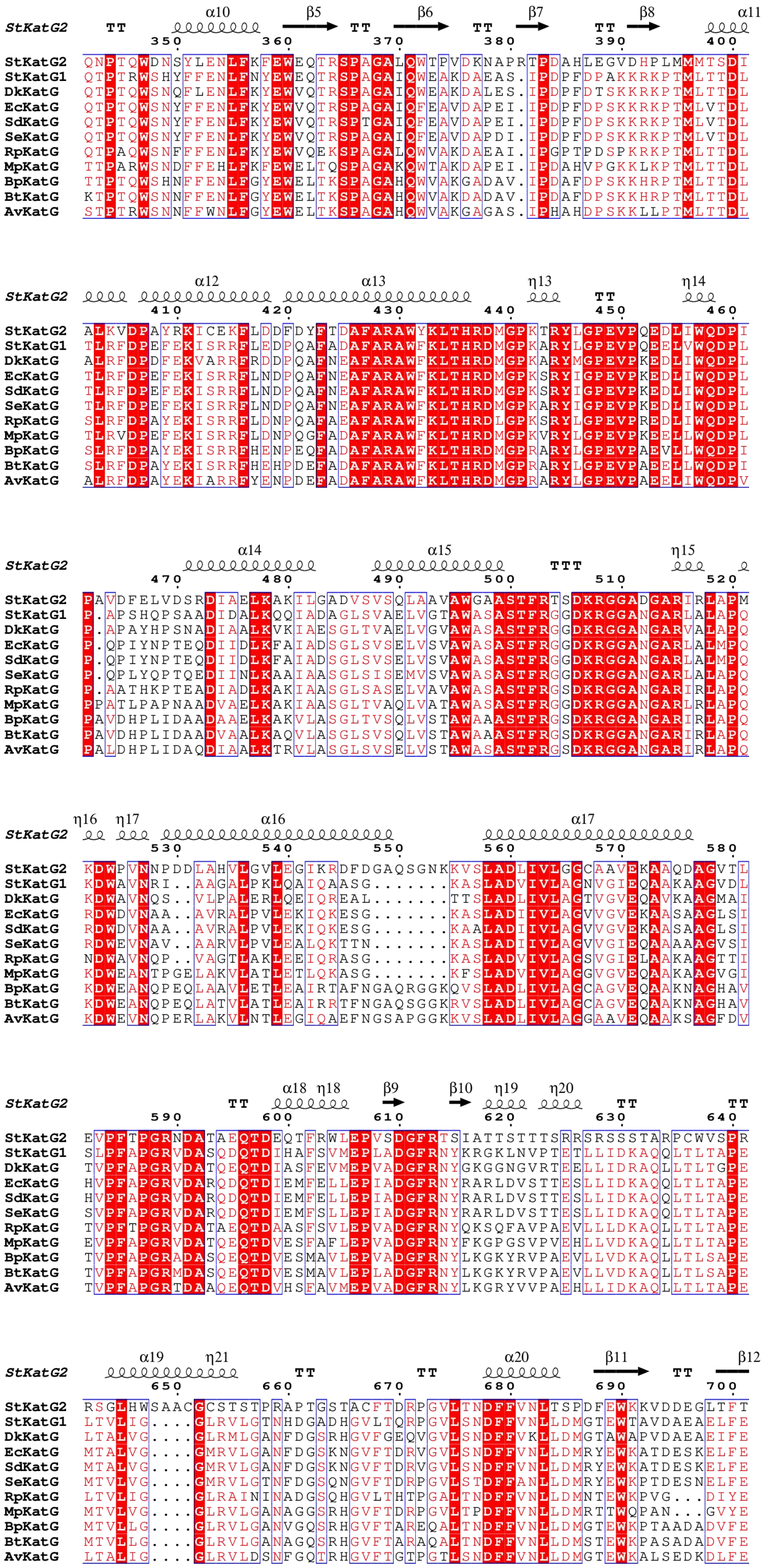
StKatG2 TT α10 β5 TT β6 TT β7 TT β8 α11
350 360 370 380 390 400
StKatG2 QNPTQWDNSYLENLFKFEWEQTRSPAGALQWTPVDKNAPRTPDAHLEGVDHPLMMTSDI
StKatG1 QTPTRWSHYFFENLFNYEWEQTRSPAGAIQWEAKDAEAS.IPDPFDPAKKRKPTMLTTDL
DkKatG QTPTQWSNQFLENLFKYEWVQTRSPAGAIQWEAKDALES.IPDPFDTSKKRKPTMLTTDL
EcKatG QTPTQWSNYFFENLFKYEWVQTRSPAGAIQFEAVDAPEI.IPDPFDPSKKRKPTMLVTDL
SdKatG QTPTQWSNYFFENLFKYEWVQTRSPTGAIQFEAVDAPEI.IPDPFDPSKKRKPTMLVTDL
SeKatG QTPTQWSNYFFENLFKYEWVQTRSPAGAIQFEAVDAPDI.IPDPFDPSKKRKPTMLVTDL
RpKatG QTPAQWSNFFFENLFKYEWVQEKSPAGALQWVAKDAEAI.IPGPTPDSPKRKPTMLTTDL
MpKatG TTPARWSNDFFEHLFKFEWELTQSPAGAKQWTAKDAPEI.IPDAHVPGKKLKPTMLTTDL
BpKatG TTPTQWSHNFFENLFGYEWELTKSPAGAHQWVAKGADAV.IPDAFDPSKKHRPTMLTTDL
BtKatG KTPTQWSNNFFENLFGYEWELTKSPAGAHQWVAKDADAV.IPDAFDPSKKHRPTMLTTDL
AvKatG STPTRWSNNFFWNLFGYEWELTKSPAGAHQWVAKGAGAS.IPHAHDPSKKLLPTMLTTDL
StKatG2 α12 α13 η13 TT η14
410 420 430 440 450 460
StKatG2 ALKVDPAYRKICEKFLDDFDYFTDAFARAWYKLTHRDMGPKTRYLGPEVPQEDLIWQDPI
StKatG1 TLRFDPEFEKISRRFLEDPQAFADAFARAWFKLTHRDMGPKARYLGPEVPQEELVWQDPL
DkKatG ALRFDPDFEKVARRFRDDPQAFNEAFARAWFKLTHRDMGPKARYMGPEVPKEDLIWQDPL
EcKatG TLRFDPEFEKISRRFLNDPQAFNEAFARAWFKLTHRDMGPKSRYIGPEVPKEDLIWQDPL
SdKatG TLRFDPEFEKISRRFLNDPQAFNEAFARAWFKLTHRDMGPKSRYIGPEVPKEDLIWQDPL
SeKatG TLRFDPEFEKISRRFLNDPQAFNEAFARAWFKLTHRDMGPKARYIGPEVPKEDLIWQDPL
RpKatG SLRFDPAYEKISRRFLDNPQAFAEAFARAWFKLTHRDLGPKSRYLGPEVPREDLIWQDPL
MpKatG TLRVDPEFEKISRRFLDNPQGFADAFARAWFKLTHRDMGPKVRYLGPEVPKEELLWQDPL
BpKatG SLRFDPAYEKISRRFHENPEQFADAFARAWFKLTHRDMGPRARYLGPEVPAEVLLWQDPI
BtKatG SLRFDPAYEKISRRFHEHPDEFADAFARAWFKLTHRDMGPRARYLGPEVPAEELLWQDPI
AvKatG ALRFDPAYEKIARRFYENPDEFADAFARAWFKLTHRDMGPRARYLGPEVPAEELIWQDPV
StKatG2 α14 α15 TTT η15
470 480 490 500 510 520
StKatG2 PAVDFELVDSRDIAELKAKILGADVSVSQLAAVAWGAASTFRTSDKRGGADGARIRLAPM
StKatG1 P.APSHQPSAADIDALKQQIADAGLSVAELVGTAWASASTFRGGDKRGGANGARLALAPQ
DkKatG P.APAYHPSNADIAALKVKIAESGLTVAELVGVAWASASTFRGGDKRGGANGARVALAPQ
EcKatG P.QPIYNPTEQDIIDLKFAIADSGLSVSELVSVAWASASTFRGGDKRGGANGARLALMPQ
SdKatG P.QPIYNPTEQDIIDLKFAIADSGLSVSELVSVAWASASTFRGGDKRGGANGARLALMPQ
SeKatG P.QPLYQPTQEDIINLKAAIAASGLSISEMVSVAWASASTFRGGDKRGGANGARLALAPQ
RpKatG P.AATHKPTEADIADLKAKIAASGLSASELVAVAWASASTFRGSDKRGGANGARIRLAPQ
MpKatG PPATLPAPNAADVAELKAKIAASGLTVAQLVATAWASASTFRGGDKRGGANGARLRLAPQ
BpKatG PAVDHPLIDAADAAELKAKVLASGLTVSQLVSTAWAAASTFRGSDKRGGANGARIRLAPQ
BtKatG PAVDHPLIDAADVAALKAQVLASGLSVSQLVSTAWAAASTFRGSDKRGGANGARIRLAPQ
AvKatG PALDHPLIDAQDIAALKTRVLASGLSVSELVSTAWASASTFRGSDKRGGANGARIRLAPQ
η16 η17 α16 α17
StKatG2
530 540 550 560 570 580
StKatG2 KDWPVNNPDDLAHVLGVLEGIKRDFDGAQSGNKKVSLADLIVLGGCAAVEKAAQDAGVTL
StKatG1 KDWAVNRI..AAGALPKLQAIQAASG.......KASLADLIVLAGNVGIEQAAKAAGVDL
DkKatG KDWAVNQS..VLPALERLQEIQREAL.......TTSLADLIVLAGTVGVEQAVKAAGMAI
EcKatG RDWDVNAA..AVRALPVLEKIQKESG.......KASLADIIVLAGVVGVEKAASAAGLSI
SdKatG RDWDVNAA..AVRALPVLEKIQKESG.......KAALADIIVLAGVVGVEKAASAAGLSI
SeKatG RDWEVNAV..AARVLPVLEALQKTTN.......KASLADIIVLAGVVGIEQAAAAAGVSI
RpKatG NDWAVNQP..VAGTLAKLEEIQRASG.......KASLADVIVLAGSVGIELAAKAAGTTI
MpKatG KDWEANTPGELAKVLATLETLQKASG.......KFSLADVIVLAGGVGVEQAAKAAGVGI
BpKatG KDWEANQPEQLAAVLETLEAIRTAFNGAQRGGKQVSLADLIVLAGCAGVEQAAKNAGHAV
BtKatG KDWEANQPEQLATVLATLEAIRRTFNGAQSGGKRVSLADLIVLAGCAGVEQAAKNAGHAV
AvKatG KDWEVNQPERLAKVLNTLEGIQAEFNGSAPGGKKVSLADLIVLAGGAAVEQAAKSAGFDV
StKatG2 TT α18 η18 β9 β10 η19 η20 TT TT
590 600 610 620 630 640
StKatG2 EVPFTPGRNDATAEQTDEQTFRWLEPVSDGFRTSIATTSTTTSRRSRSSSTARPCWVSPR
StKatG1 SLPFAPGRVDASQDQTDIHAFSVMEPLADGFRNYKRGKLNVPTETLLIDKAQQLTLTAPE
DkKatG TVPFAPGRVDATQEQTDIASFEVMEPVADGFRNYGKGGNGVRTEELLIDRAQLLTLTGPE
EcKatG HVPFAPGRVDARQDQTDIEMFELLEPIADGFRNYRARLDVSTTESLLIDKAQQLTLTAPE
SdKatG HVPFAPGRVDARQDQTDIEMFELLEPIADGFRNYRARLDVSTTESLLIDKAQQLTLTAPE
SeKatG SVPFAPGRVDARQDQTDIEMFSLLEPIADGFRNYRARLDVSTTESLLIDKAQQLTLTAPE
RpKatG TVPFTPGRVDATAEQTDAASFSVLEPVADGFRNYQKSQFAVPAEVLLLDKAQLLTLTAPE
MpKatG EVPFAPGRVDATQEQTDVESFAFLEPVADGFRNYFKGPGSVPVEHLLVDKAQLLTLTAPE
BpKatG TVPFAPGRADASQEQTDVESMAVLEPVADGFRNYLKGKYRVPAEVLLVDKAQLLTLSAPE
BtKatG TVPFAPGRMDASQEQTDVESMAVLEPLADGFRNYLKGKYRVPAEVLLVDKAQLLTLSAPE
AvKatG TVPFAPGRTDAAQEQTDVHSFAVMEPVADGFRNYLKGRYVVPAEHLLIDKAQLLTLTAPE
StKatG2 α19 η21 TT TT α20 β11 TT β12
650 660 670 680 690 700
StKatG2 RSGLHWSAACGCSTSTPRAPTGSTACFTDRPGVLTNDFFVNLTSPDFEWKKVDDEGLTFT
StKatG1 LTVLIG....GLRVLGTNHDGADHGVLTQRPGVLSNDFFVNLLDMGTEWTAVDAEAELFE
DkKatG LTALVG....GLRMLGANFDGSRHGVFGEQVGVLSNDFFVKLLDMGTAWAPVDAEAEIFE
EcKatG MTALVG....GMRVLGANFDGSKNGVFTDRVGVLSNDFFVNLLDMRYEWKATDESKELFE
SdKatG MTALVG....GMRVLGANFDGSKNGVFTDRVGVLSNDFFVNLLDMRYEWKATDESKELFE
SeKatG MTVLVG....GMRVLGTNFDGSQNGVFTDRPGVLSTDFFANLLDMRYEWKPTDESNELFE
RpKatG LTVLIG....GLRAININADGSQHGVLTHTPGALTNDFFVNLLDMNTEWKPVG...DIYE
MpKatG MTVLVG....GLRVLGANAGGSRHGVFTDRPGVLTPDFFVNLLDMRTTWQPAN...GVYE
BpKatG MTVLLG....GLRVLGANVGQSRHGVFTAREQALTNDFFVNLLDMGTEWKPTAADADVFE
BtKatG MTVLLG....GLRVLGANAGQSRHGVFTARAQALTNDFFVNLLDMSTEWKPASADADVFE
AvKatG LTALIG....GLRVLDSNFGQTRHGVFTGTPGTLSNDFFVNLLDMGTEWKALSEDKDLFE

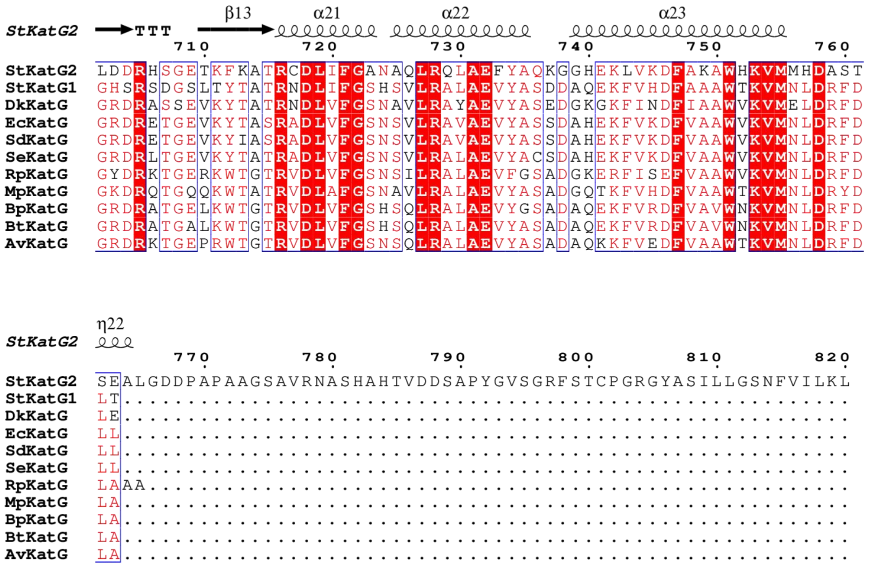

Figure S1. Sequence alignment of full-length StKatG2 and other catalase-peroxidases. The same sequences analysis with Fig. 1 (with the exception that Fig. 1 shows partial-length protein sequences). The sequences were aligned using the Clustal Omega and treated with ESPript 3 (https://espript.ibcp.fr/). The secondary structure elements presented on top were obtained from the predicted StKatG2 structure using Alphafold3 (α, α-helices; η, 3 10 helices; β, β-strands. TT, turns). Identical and similar residues are displayed in red and blue boxes, respectively. StKatG1 and StKatG2, *Salinicola tamaricis* KatGs; DkKatG, *Drosophila kikkawai* KatG; EcKatG, *Escherichia coli* KatG; SdKatG, *Shigella dysenteriae* KatG; SeKatG, *Salmonella enterica* KatG; RpKatG, *Ralstonia pickettii* KatG; MpKatG, *Methylibium petroleiphilum* KatG; BpKatG, *Burkholderia pseudomallei* KatG; BtKatG, *Burkholderia thailandensis* KatG; AvKatG, *Azotobacter vinelandii* KatG.

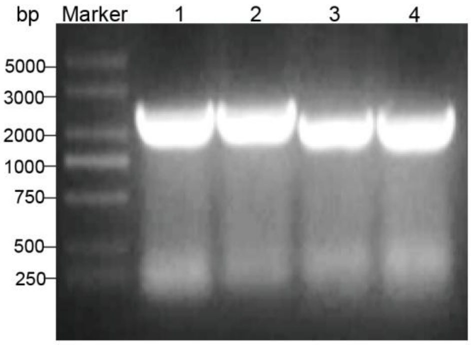

Figure S2. Verification of the heterologous expression vectors containing StKatG1 and StKatG2. Lane 1, 2: amplification of the *stkatG1* gene from the recombinant plasmid, resulting in a product of 2196 bp in length; Lane 3, 4: amplification of the *stkatG2* gene from the recombinant plasmid, yielding a product of 2297 bp. Marker: molecular weight marker.

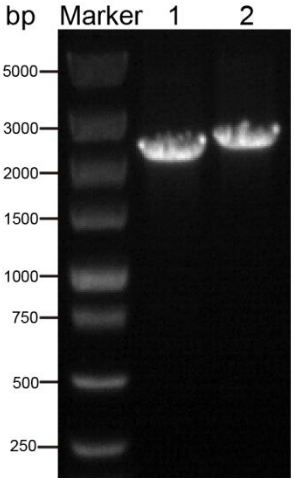

Figure S3. DNA agarose gel electrophoresis of *stkatG1* and *stkatG2*. Lane 1: PCR amplified *stkatG1* gene (2196 bp) product; Lane 2: PCR amplified *stkatG2* gene (2297 bp) product. Marker: molecular weight marker.

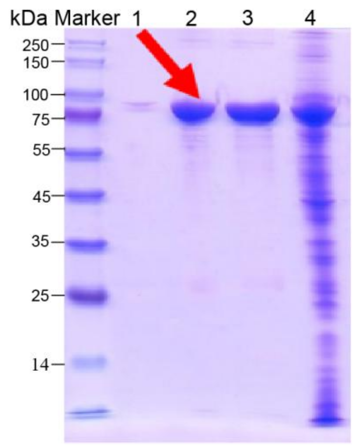

Figure S4. SDS-PAGE analysis of the purified recombinant StKatG2. The StKatG2 protein has an approximate molecular weight of 85 kDa, as indicated by the red arrow. Wash and elute fractions corresponding to StKatG2 were loaded onto 10% SDS-PAGE gel. Lane 1: the protein in wash buffer after passing nickel purification column; Lane 2: purified protein in Tris-HCl buffer; Lane 3: purified protein in elute buffer; Lane 4: crude cell extract before subjecting onto the nickel purification column. Marker: molecular weight marker.

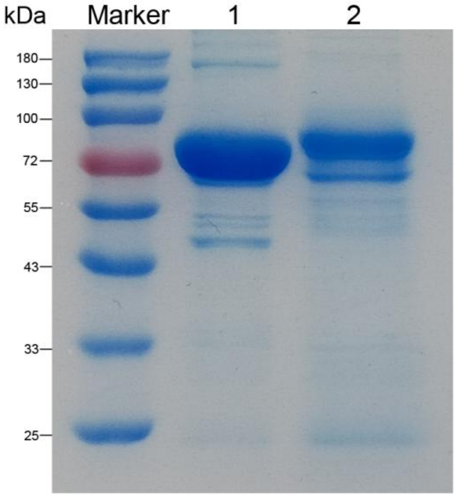

Figure S5. SDS-PAGE analysis of the purified recombinant StKatG1 and StKatG2. The proteins were loaded onto 10% SDS-PAGE gel. Lane 1: purified StKatG1; Lane 2: purified StKatG2. Marker: molecular weight marker.

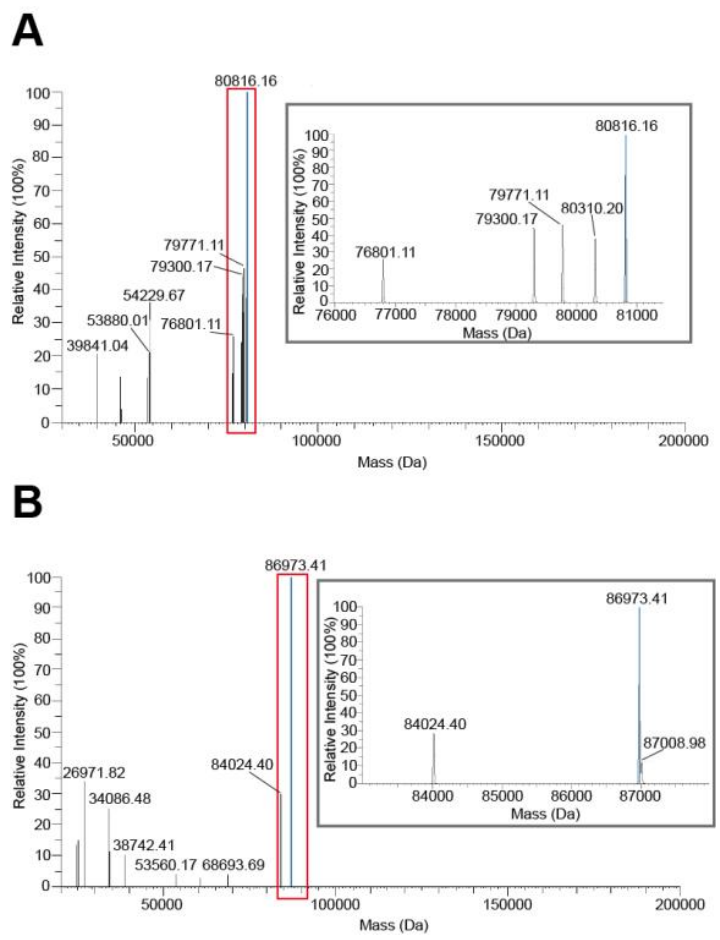

Figure S6. Mass spectrometry profiles of purified enzymes. Analyses were conducted using HPLC-MS. The *m/z* spectra were converted into molecular mass profiles. (A) The mass spectrometry profile of purified StKatG1, with the upper right image displaying a magnified view of the molecular weight range from 76.0 to 81.8 kDa, highlighted within a red box. (B) The mass spectrometry profile of purified StKatG2, with the upper right image providing a magnified view of the molecular weight range from 83.8 to 87.5 kDa, also highlighted within a red box.

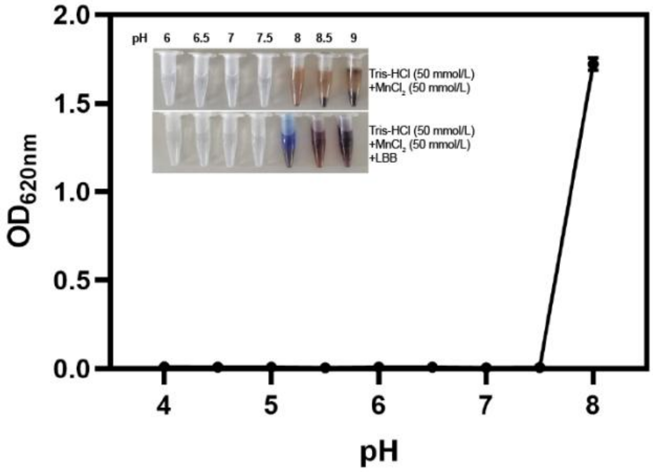

Figure S7. Manganese autooxidation system without enzyme in Tris-HCl (50 mmol/L). The autoxidation of Mn(II) occurred when the pH value exceeded 8.0. The images of the reaction system is derived from our previous study (Zhao et al., 2023). Up, $MnCl_2$ (50 mmol/L) was incubated for 24 h at 30ºC in Tris-HCl (50 mmol/L) with different pH values. Down, 300 µL of each sample from A was reacted for 2 h at 30°C with 60 µL of LBB and 900 µL of acetic acid (45 mmol/L).

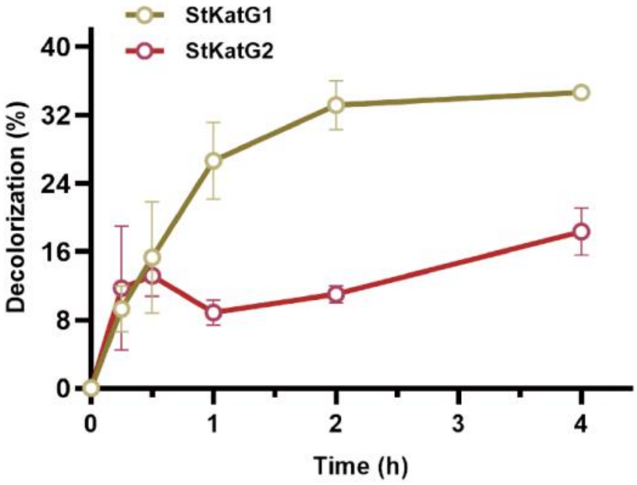

Figure S8. Decolorization experiment of StKatG1 and StKatG2 on MG with a concentration at 80 mg/L.

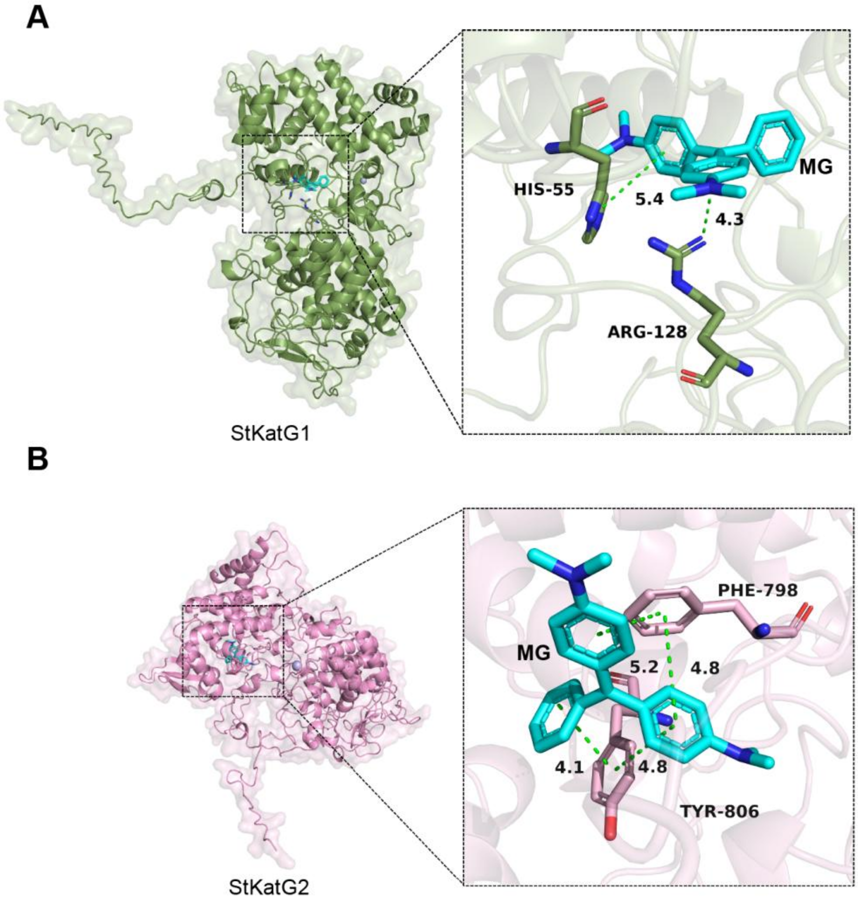

Figure S9. Docking analysis of StKatG1 and StKatG2 with MG. (A) Molecular docking between StKatG1 and MG, where HIS55 and ARG128 establish hydrophobic interaction with MG. (B) Molecular docking between StKatG2 and MG, in which PHE798 and TYR806 form hydrophobic interaction with MG. The manganese binding site in the protein is represented as a purple sphere, while MG is illustrated as a blue compound structure.

Table S1. HPLC-MS analysis of StKatG1 protein and detailed table of protein molecular weight analysis.

Average Mass (kDa)	Intensity	Relative Abundance (%)	Fractional Abundance	Score	Number of Charge States	Charge State Distribution	Mass Std Dev	Delta Mass	Start Time (min)	Stop Time (min)
80816.16	1.05E+06	100.00	26.28	111.02	20	61 - 80	6.02	0.00	7.509	10.037
79771.11	4.98E+05	47.44	12.47	53.74	9	70 - 78	5.31	-1045.05	7.509	10.037
79300.17	4.82E+05	45.95	12.08	52.57	9	71 - 79	5.74	-1515.98	7.509	10.037
80310.20	3.88E+05	37.00	9.72	66.14	11	70 - 80	6.11	-505.96	7.509	10.037
54229.67	3.62E+05	34.50	9.07	60.76	11	43 - 53	4.07	-26586.49	7.509	10.037
95716.05	2.63E+05	25.04	6.58	48.40	9	81 - 89	11.20	14899.90	7.509	10.037
53880.01	2.28E+05	21.72	5.71	46.18	8	46-53	3.50	-26586.49	7.509	10.037
39841.04	2.20E+05	20.97	5.51	44.11	8	31-38	3.25	-40975.12	7.509	10.037
76801.11	2.13E+05	20.3	5.34	40.95	8	63-70	6.01	-4015.05	7.509	10.037
46179.12	1.47E+05	13.97	3.67	43.13	8	35-42	2.09	-34637.04	7.509	10.037
53429.23	1.43E+05	13.59	3.57	44.09	8	37-44	4.65	-27386.93	7.509	10.037

Table S2. HPLC-MS analysis of StKatG2 protein and detailed table of protein molecular weight analysis.

Average Mass (kDa)	Intensity	Relative Abundance (%)	Fractional Abundance	Score	Number of Charge States	Charge State Distribution	Mass Std Dev	Delta Mass	Start Time (min)	Stop Time (min)
86973.40	1.23E+06	100.00	40.30	134.48	23	77 - 99	3.05	0.00	8.217	9.642
26971.82	4.17E+05	33.89	13.66	45.83	9	23 - 31	1.01	-60001.59	8.217	9.642
84024.40	3.69E+05	29.98	12.08	95.90	18	72 - 89	4.34	-2949.01	8.217	9.642
34086.48	3.09E+05	25.14	10.13	64.25	12	29 - 40	2.07	-52886.93	8.217	9.642
25098.02	1.85E+05	15.01	6.05	40.92	9	21 - 29	1.00	-61875.39	8.217	9.642
24697.23	1.65E+05	13.44	5.42	34.85	7	23 - 29	1.10	-62276.17	8.217	9.642
38742.41	1.26E+05	10.27	4.14	42.86	7	39 - 45	1.69	-48231.00	8.217	9.642
87007.98	1.22E+05	9.93	4.00	64.29	11	83 - 93	4.83	34.58	8.217	9.642
68693.69	4.83E+04	3.92	1.58	46.72	8	58 - 65	3.73	-18279.72	8.217	9.642
53560.17	4.73E+04	3.84	1.55	37.54	7	50 - 56	3.32	-33413.24	8.217	9.642
60657.35	3.36E+04	2.73	1.10	42.55	7	52 - 58	3.24	-26316.05	8.217	9.642

Table S3. Molecular and biochemical differences between the StkatG1 and StkatG2.

Molecular and biochemical properties		StKatG1[a]	StKatG2
Gene length		2196 bp	2297 bp
Protein molecular weight		80.82 kDa	86.97 kDa
Optimal pH		7.5	7.5
Optimal temperature		50°C	55°C
Thermostability (50°C)		82.3%	73.6%
Morphology of BioMnOx		Layered sheet-like structure	Spherical or irregular structure
Composition of BioMnOx		MnO_2、Mn_3O_4、C_2MnO_4	MnO_2、Mn_3O_4、C_2MnO_4
Oxidation states of Mn on BioMnOx		$Mn2p_{1/2}$、$Mn2p_{2/3}$	$Mn2p_{1/2}$、$Mn2p_{2/3}$
Degradation efficiency of MG	20 mg/L	63.91%	73.38%
	50 mg/L	51.78%	60.08%

[a] Footnote: These data are from previous studies (Zhao et al., 2023).

Reference

Zhao, G., Wang, W., Zheng, L., Chen, L., Duan, G., Chang, R., et al. (2023). Catalase-peroxidase StKatG is a bacterial manganese oxidase from endophytic Salinicola tamaricis. *Int J Biol Macromol* 224**,** 281-291. doi: 10.1016/j.ijbiomac.2022.10.123.
